# Psychological Quality of Life in People with Physical Disability: The Effect of Internalized Stigma, Collective Action and Resilience

**DOI:** 10.3390/ijerph17051802

**Published:** 2020-03-10

**Authors:** Prado Silván-Ferrero, Patricia Recio, Fernando Molero, Encarnación Nouvilas-Pallejà

**Affiliations:** Faculty of Psychology, National University for Distance Education (UNED), 28040 Madrid, Spain; mdpsilvan@psi.uned.es (P.S.-F.); reciop@psi.uned.es (P.R.); fmolero@psi.uned.es (F.M.)

**Keywords:** resilience, internalized stigma, collective action, disability, quality of life

## Abstract

Purpose: The main objective of this study was to examine the role of social identification, collective action and resilience in reducing the negative consequences of internalized stigma on the psychological quality of life of people with physical disability using path analysis. We propose a model with two paths: the first through social identification and collective action and the second via resilience. Method: A total of 288 Spanish people with physical disability aged between 18 and 82 years (46.4% males; mean [SD] of age = 45.1 [12.3] responded to the questionnaire. Data were collected for three months through an online survey. Results: The tested model adequately fit the data. We found that the relationship between internalized stigma and the psychological quality of life of people with physical disability was mediated by resilience. However, neither social identification nor collective action mediated the association between internalized stigma and quality of life among our participants. Conclusions: The results confirmed the negative association between internalized stigma and quality of life in the population with physical disability. The results show that some interactive processes, such as resilience, may contribute to decreasing the negative effects of internalized stigma. In contrast, no effects of identification with the group or collective action intention were found.

## 1. Introduction

In 1963, Goffman [1] defined stigma as a discrediting attribute that places a subject into a different and undesirable category. From this initial conceptualization, the definition of this term has evolved to highlight the importance of the situational component in addition to observable characteristics. Another definition highlights the role of a specific social context in devaluating a stigmatized person [2]. That is, it is necessary to consider the context in which stigmatization occurs in addition to observable physical characteristics. [3] defined stigma as “a social construction” that includes the recognition of a difference based on a branding and the subsequent devaluation of a person. Stigma assumes the existence of a negative status established in society and negative attitudes that lead to discriminatory behaviour towards a stigmatized group [4,5].

Previous research reveals that people with disability in their daily living may encounter unfriendly treatment and are at risk of being stigmatized, e.g., [6,7]. Stigma towards this group can be reflected in different ways, such as the experience of discomfort or anxiety in social interactions with this group [8] or in negative stereotypes in which people with disability are different from fully human people [9]. Perceived discrimination may have negative effects on the well-being of people with disability [10], but the worst consequence is the internalization of stigma [11].

Internalized stigma is defined as the acceptance of stigma by a stigmatized person as part of the person’s own system of values and self-concept [12]. Studies on the groups with high levels of stigma, such as people with mental illness [13,14,15,16,17,18,19,20] or family members of people with mental illness [21,22,23,24,25,26], children with learning disabilities [27], people with epilepsy [28], people with weight concerns [29,30,31,32,33,34,35,36,37,38,39], and people with HIV [40,41,42] highlight the highly negative effects of internalized stigma. However, so far, research on the internalized stigma of people with disability and its consequences is scarce. Recent research has shown the mediating effect of internalized stigma on the relationship between perceived personal discrimination and self-esteem in people with disability [43].

The negative impact of internalized stigma on quality of life has been found in some previous research examining stigmatized groups. In the case of mental illness, the link between internalized stigma and quality of life has been well established. For example, [44] found a theoretical model of the relationship between internalized stigma and quality of life, which was mediated by a sense of coherence and self-esteem. Similarly, [45] showed that internalized stigma is a predictor of lower quality of life in different areas of life (work/occupation, leisure time and mental health) in outpatients with bipolar disorder.

The main goal of this paper is to examine the role of two different strategies that can reduce the negative consequences of internalized stigma for the psychological quality of life in people with physical disability in Spain using structural equation modelling. The first path is through group identification and collective action, and the second path is via resilience to promote a positive psychological quality of life in people with disability.

### 1.1. The Rejection-Identification Model

As stated before, the first path we propose to reduce the negative influence of internalized stigma is via group identification and collective action. The rejection-identification model (RIM) [46] posits that perceived group discrimination increases in-group identification, which in turn prevents some of the negative effects of discrimination. Thus, social identification can play a protective role between perceived discrimination and its negative consequences, as has been shown in different groups [46,47,48,49,50]. However, the RIM has received incomplete support in some studies that differentiate between individual and group discrimination [51,52,53], while other studies provide no support for the model [54,55].

Previous research points out that group identification can be a precondition for collective action [56,57,58]. Thus, members who identify more strongly with their group are more likely to engage in collective action in order to improve their status. The association between group identification and collective action is supported by ample evidence in various stigmatized groups [11,59,60,61,62]. In people with several types of disability, group identification was found to predict involvement in political organizations with the aim to improve their status [63]. However, there is no clear evidence of the influence that collective action may have on well-being. Positive effects were found in people with HIV [64] and in lesbians and gay people [62], but for people with mental illness, collective action was positively and negatively associated with well-being [11].

In this research, we examine disability from a social identity perspective [59] considering social identification as a precondition for collective action. Based on this theoretical framework, we expect that internalized stigma may enhance group identification, which in turn may strengthen collective action tendencies. That is, the link between group identification and collective action may reduce the negative effects of internalized stigma on psychological quality of life.

### 1.2. Resilience, Stigma and Quality of Life

Resilience, as a theoretical construct, is difficult to conceptualize. A classic definition considers resilience a “universal capacity which allows a person, group or community to prevent, minimize or overcome the damaging effect of adversity” [65], p. 4. Thus, resilience supports the development of social, academic and emotional competence despite exposure to stressful situations and substantial difficulties.

Researchers use this concept when referring to a socially competent person with identity consciousness who is able to make decisions; has aims and life goals; can satisfy his or her basic affect, relationship and respect needs and can grow through adversity and disruption [66,67,68,69]. However, resilience is not a trait but an interactive process: It is a set of intrapsychic and social processes that occur over time by combining personal attributes and the social and cultural environment [69,70,71,72]. Thus, [73] defined resilience as “the role of mental processes and behaviour in promoting personal assets and protecting an individual from potential negative effects of stressors” (p. 16).

Resilience studies are fundamentally linked to adversity, as the main antecedent, and positive adaptation, as the main consequence [73]. For example, some authors have considered that resilience in the adversity context is dynamic and encompasses positive adaptation [74,75]. Davidov and his collaborators (2013) noted that resilience can be defined as synonymous with reduced vulnerability, with the ability to adapt to or cope with adversity [76,77,78]. However, it is important to note the distinction between resilience and coping [74]. For example, in Lazarus and Folkman’s (1984) definition [79], coping is conceived as the efforts made by a person “to manage specific external demands that are appraised as taxing or exceeding the person’s resources” (p. 141). Thus, resilience is involved in a person’s evaluation of a stressful event, whereas coping refers to the strategies that a person employs to face a stressful event. Furthermore, resilience is a response with a positive direction, whereas this is not necessarily the case with coping strategies.

The effects of protective and promotive factors vary contextually and temporally [66,74,80]. The sociocultural context is important when examining positive adaptation, and it can change throughout a situation and across an individual’s lifespan. With respect to time, there are significant differences between age groups in favour of older people versus middle-aged and young people [81].

Given the importance of the interaction between people and their environment, we should understand the important role of resilience in the stress process. Lazarus’s cognitive-motivational-relational theory (1991) [79] and, subsequently, a meta-model on stress, emotion, and performance proposed by Fletcher and Scott [82] describes how an individual reacts to stressful situations. The model also explains how an individual’s action affects his/her performance [73,74,83] with the conceptualization that stressors increased in the individual’s environment. Specifically, processes of perception, appraisal and coping are potential mediators given that they could result in positive or negative responses and different types of feeling states. There are several situational and personal characteristics (positive affect, self-efficacy, self-esteem) that moderate the ongoing process. Specifically, “resilience-related variables can influence the stress process in the individual appraisal of stressors, his meta-cognitions and his selection of coping strategies” (p. 16). In fact, it has been shown that a higher degree of resilience is associated with better quality of life and a lower degree of internalized stigma in patients with schizophrenia [84,85,86]. In bipolar patients, resilience is a predictor of overall quality of life [45]. In general, it is believed that the psychosocial aspects associated with resilience explain this association [87].

People with disability (motor or sensory) are in a difficult situation: they face barriers in their daily activities and have an increased risk of developing various symptoms, stigma and secondary maladies (e.g., fatigue, depression, pain). It is necessary to identify protective factors that could help these people function healthily and improve their quality of life despite these barriers [88]. Resilience may play an important role for people who encounter many difficult barriers in their everyday life functioning [89].

Although little work has focused on the role of resilience in adult people with disability [66,69,88,89,90,91], resilience can produce some responses that demonstrate the relevance of this factor in difficult situations. A study found that depression was significantly associated with lowered resilience among people with disability [91]. Another study found that a decrease in resilience was associated with an increase in depression and fatigue, whereas an increase in resilience was associated with sleep quality and physical functioning in ageing people with disability [89]. However, [88] found that physical functioning was not strongly influenced by resilience.

These different results show the need for more research to clarify how resilience can influence the way that people with disability face their situation and overcome barriers to experience a better quality of life.

### 1.3. Goals and Hypotheses

**Figure 1:**
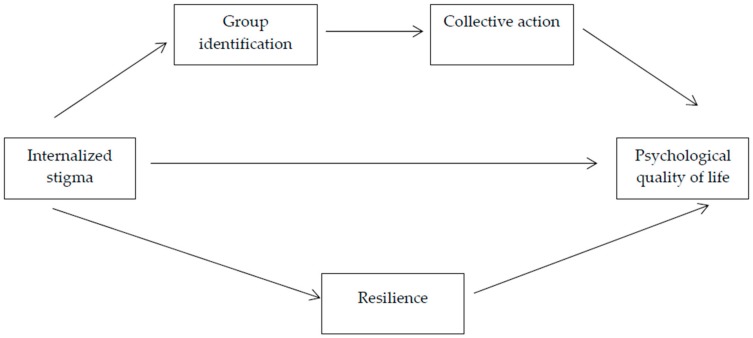
Model of psychological quality of life in people with disability.

The present research aims to examine the consequences of internalized stigma for the psychological quality of life of people with physical disability using path analysis. We propose a model (Figure 1) in which internalized stigma is related to psychological quality of life by means of two pathways that may constitute two ways to cope with stigma. The first path is collective/external in nature, and the second is of an internal nature.

In the current study, we examine whether group identification, collective action and resilience mediate the association between internalized stigma and psychological quality of life of people with physical disability. As shown in Figure 1, we hypothesize a multiple mediation model in which we expect that group identification and collective action mediate the association between internalized stigma and psychological quality of life. We further hypothesize that resilience mediates the relation between internalized stigma and psychological quality of life.

## 2. Method

### 2.1. Participants

The participants were 289 Spanish people (46.4% men and 53.3% women) with physical disability. Their age ranged between 18 and 82 years (*M* = 45.1; *SD* = 12.3). All participants had their disability certified according to the Spanish administration’s Royal Decree law 1971/1999 of December 23. Among the participants, 72% were between 33% and 65% disabled (which allowed them to obtain a Disability Certificate that gives them access to some benefits, rights and services), and 28% were more than 65% disabled (which means they qualified for a non-contributory pension). Most had secondary education or vocational training (47.9%); the rest had higher education (26.9%), primary education (22.7%) or no formal education (2.4%).

### 2.2. Measures

**Stigma scale for chronic illness 9-item version (SSCI-9).** The Spanish adaptation of the internalized stigma subscale of the SSCI [92], which had shown adequate psychometric properties in a of people with different disabilities, was used for the research [93,94]. Example items are “*Because of my illness, I feel emotionally distant from other people*” and “*Because of my illness, I feel embarrassed in social situations*”. Each item is rated on a four-point scale that ranges from 1 (never or almost never) to 4 (always or almost always), and total scores range from 9 to 36. Higher scores indicate higher levels of stigma. In our study, the scale showed good internal consistency, with a Cronbach’s alpha value of 0.91 and a composite reliability (CR) value of 0.91.

**Group identification.** We measured identification using the Spanish version [60] of a previously validated six-item measure [95]. Participants responded to each item by indicating the degree to which they agreed with it on a 4-point Likert scale. Sample items are “*When someone criticizes people with my same disability, it feels like a personal insult” and “I am very interested in what others think about people with disability*”. The alpha coefficient for this scale was 0.82, and CR was 0.85.

**Collective action.** Four items were used to assess the collective action, including its perceived effectiveness and the intention to perform it [62]. An example item is “*I am willing to participate in collective actions to support the rights of people with disability*”. Participants were requested to respond on a 4-point Likert scale ranging from 1 (no agreement) to 4 (totally agree). In the current study, the internal consistency results showed good reliability (α= 0.80 and CR = 0.81).

**Resilience.** The 10-item Conner–Davidson Resilience Scale was used to assess resilience [96], a self-administered questionnaire with a Likert-type additive scale containing four response options (from 0 = never to 4 = almost always). The original version had a single dimension. Example items are “*Under pressure, I focus and think clearly*” and “*I am not easily discouraged by failure*”. We used the Spanish version [97], which showed adequate reliability in our sample (α= 0.90 and CR = 0.92).

**Psychological quality of life.** We used the subscale of the psychological domain of the Spanish adaptation of the short version of the WHO quality of life questionnaire (WHOQOL-BREF) [98,99]. Example items are “*How much do you enjoy life*?” and “*To what extent do you think your life makes sense?*” The six items that composed the subscale were answered on a five-point scale, with high scores representing higher quality of life in the psychological domain (with item 26 reverse coded). In our study, the scale showed good internal consistency (Cronbach’s alpha = 0.80 and CR = 0.83).

### 2.3. Procedure

Undergraduate students majored in psychology in a Spanish university (i.e., Universidad Nacional de Educación a Distancia (UNED; National Open University)) assisted in participants recruitment personally. The students identified the target participants (i.e., those with physical disability) and explained study objections together with instructions for questionnaire completion to them. The collection period was three months. Participants were guaranteed anonymity and confidentiality. Once they had registered and filled in the consent form, participants completed the questionnaire for approximately 20–30 min online using Qualtrics software. The research was previously approved by the University Ethics Committee and developed following the ethical standards of the Declaration of Helsinki.

### 2.4. Statistical Analysis

First, a preliminary analysis was carried to determine the descriptive statistics and correlations between the variables in order to understand the basic properties and the relationships between the variables. Next, path analysis was used to test the proposed model using AMOS 24 (IBM CORP, Lincoln, NE, USA). Model estimations used maximum likelihood estimation, so multivariate normality was statistically evaluated by critical ratio of multivariate kurtosis proposed by Mardia, where a critical ration below suggests multivariate normality. A critical ratio of kurtosis <5.0 indicates multivariate normality [100]. We found a Mardia’s coefficient equal to −0.87; therefore, our data showed a multivariate normal distribution. Calculation of the Mahalanobis distance revealed no multivariate outliers in the sample. The indexes of fit included χ^2^/df (good fit <3) and the Root Mean Square Residual (RMR), both used as indexes of absolute fit (there is a good model fit if RMR is less than or equal to 0.05, and there is adequate fit if RMR is less than 0.08). The Normed Fit Index (NFI) and Comparative Fit Index (CFI) were used as indexes of incremental fit, both ranging from 0 to 1; values between 0.90 and 0.95 indicate an acceptable model fit, and values greater than 0.95 indicate a close model fit [101].

Finally, a bias-corrected bootstrapping procedure was used to assess mediation effects. Mediation assessing through bootstrap method is advocated as the best approach because of its reliable estimates and valid confidence intervals for both direct and indirect effects. Through random sampling method, 10,000 resamples were generated from the original dataset. A significantly indirect effect in the bootstrapping method has its 95% confidence interval not cover zero. After comparing the significance of direct effect with and without mediators, a total mediation “(i.e., direct effect becomes nonsignificant) or a partial mediation (i.e., direct effect keeps significant).”

With the percentage of missing values below 2%, imputation is not necessary, and the final sample size used in the structural equation modelling was 275. The sample size is sufficient in providing accurate estimates, standard errors, and fit statistics [102]. Although there are no definitive rules concerning the necessary sample size for SEM techniques [103], most researchers suggest a minimum of 100–200 cases, or 10 cases per parameter estimate [104]. Our model contained 17 parameters to be estimated, so our final sample (n = 275) was adequate.

## 3. Results

Table 1 shows the descriptive statics of the studied variables and their bivariate correlation coefficients. We assessed the associations between internalized stigma, group identification, collective action, resilience and psychological quality of life. All correlations were significant except for the relationships between group identification and resilience, between group identification and psychological quality of life and between internalized stigma and collective action.

### 3.1. Model Testing

**Figure 2:**
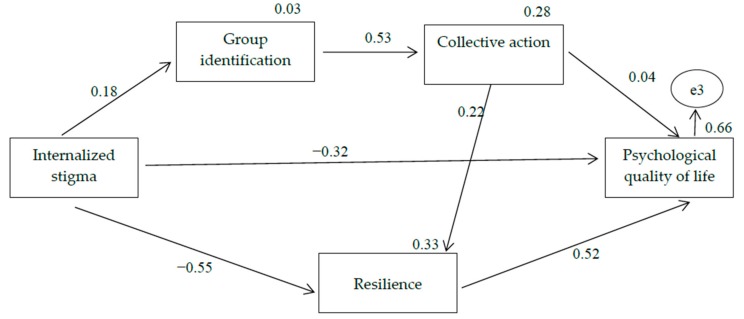
Standardized regression coefficients of the proposed model. Note: The percentage of explained variance is indicated in italics above the variable. All direct effects were significant (*p* < 0.01), except β = 0.04 (*p* = 0.31).

The results showed that the hypothesized model fit the data reasonably well, except those related to χ^2^ value, which are greatly influenced by the sample size [51]: χ^2^ = 25.52, df = 4, CFI = 0.951, NFI = 0.944 and RMR = 0.047. Modification index shown by AMOS recommended adding a path between activism and resilience to the model. After considering the theoretical feasibility of this change and verifying that there is a significant correlation between both variables (see Table 1), this change was introduced in the model and the analysis was carried out again. The final model can be seen in Figure 2. Overall, the model presented an excellent model fit to the data: the χ^2^ value was not significant (χ^2^ = 5.79, *p* = 0.122), CFI was 0.994, NFI was 0.987 and RMR was 0.019.

As shown in Figure 2, internalized stigma was positively related to group identification (β = 0.18, *p* = 0.002), which in turn was related to activism (β = 0.53, *p* < 0.001). However, collective action did not predict psychological quality of life (β = 0.04, *p* = 0.310). Collective action was positively related to resilience (β = 0.22, *p* < 0.001). On the other hand, as we hypothesized, internalized stigma was negatively related to resilience (β = −0.55, *p* < 0.001), which in turn was positively related to psychological quality of life (β = 0.52, *p* < 0.001).

### 3.2. Mediation Analyses

There was no mediating effect of the variables of group identification and collective action on the relationship between internalized stigma and psychological quality of life because there was no relationship between activism and psychological quality of life (β = 0.04, *p* = 0.310). As shown in Table 2, we found a significant indirect effect of internalized stigma on resilience via group identification and collective action (*β* = 0.021, *p* < 0.001; 95% CI: 0.008, 0.043). However, there was no mediating effect because the relationship between internalized stigma and resilience did not decrease when group identification and collective action were introduced into the model. Furthermore, there was no mediating effect of group identification on the relationship between internalized stigma and collective action because there was no direct relationship between the two variables (*β = −0*.010, *p* = 0.859), so there was no reason to check the mediating effect.

There was an indirect relationship between internalized stigma and psychological quality of life mediated by resilience. To analyse the type of mediation, we restricted the paths from internalized stigma to resilience and from resilience to psychological quality of life to 0 in the direct model. In this case, the direct relationship between internalized stigma and psychological quality of life was *β* = *−0*.60 (*p* < 0.001), and it decreased to β = −0.32 (*p* < 0.001) when resilience was introduced into the model (partial mediation). The bootstrapping results revealed that the mediating effect of resilience produced an indirect relationship (β = –0.293, *p* < 0.001; 95% CI: −0.223, −0.371).

## 4. Discussion

The main objective of this research was to examine the consequences of internalized stigma for the psychological quality of life of people with physical disability. In our proposed model, internalized stigma was related to psychological quality of life by means of two pathways that may constitute two ways to cope with stigma: first, through group identification and collective action; second, through resilience.

The results showed, first, that the proposed model offered an adequate fit to the data. Second, the relationship between internalized stigma and the psychological quality of life of people with physical disability was partially mediated by resilience. However, social identification and collective action were not mediators in the relationship between internalized stigma and quality of life.

Our results confirm the negative association between internalized stigma and quality of life in people with physical disability. This association, which has previously been found in other stigmatized groups, such as people with mental illness [44,45], can also be applied to people with physical disability.

Our data also show the mediating role of some interactive processes, such as resilience, in reducing the negative effects of internalized stigma on psychological quality of life. Previous longitudinal studies have shown that resilience is a factor that promotes successful ageing in people with physical disability [105]. In relation to motor disability, it is possible to identify different profiles of resilience, and low resilience profiles are related to a deficit in psychological adjustment and quality of life in general [69,106]. In fact, previous studies have shown the mediation effect of resilience on the demands associated with living with disability on quality of life both in adolescents with disability and their parents [64] as well as its moderating role in the relationship between affective disorders and quality of life in young adults with multiple sclerosis [107]. Although some research finds no influence of resilience on physical functioning [88], our study shows that this variable can reduce the negative effects of internalized stigma on the psychological quality of life of people with disability.

Although our data show that internalized stigma enhances group identification, which in turn promotes collective action, this link between group identification and collective action does not mediate the relationship between internalized stigma and psychological quality of life. However, in our sample, although group identification increased collective action tendencies, this did not seem to have protective effects on psychological quality of life. Research on other groups [62] has revealed the positive effect of collective action tendencies on well-being and on self-esteem in people with disability [75]. Despite this, collective action associates positively with resilience in our sample. Previous research [107] have explored the moderator role of resilience and collective action on psychological distress in a transgender sample, finding no moderation effects. More studies addressed on these relations is needed.

### 4.1. Practical Implications

From our results, we can conclude that to promote a better psychological quality of life for people with physical disability, the strengths developed through coping with daily barriers contribute to a greater extent than other variables, such as the perceived effectiveness of collective action and the intention to engage in it.

The disability paradox [108] emphasizes the importance of personal experience with disability in defining people’s self, their view of the world, their social context and their social relationships. These personal experiences may explain why people with a persistently serious disability report satisfactory quality of life even though other external observers consider that they may have an unfavourable living situation. [109].

In the same vein as the disability paradox, in our sample, participants reported good levels of psychological quality of life and resilience. Moreover, resilience reduced the negative effects of internalized stigma. This means that disability itself did not decrease a disabled individual’s quality of life and that an individual’s capacity to cope with everyday life may also be of great importance [61,110].

Our results show the need to implement intervention programmes aimed at improving the psychological quality of life of people with disability by promoting resilience. This can be achieved in two ways; first, developing activities that promote personal resources to allow good adaptation to disability and, second, encouraging people with disability to apply coping strategies adapted to the concrete context of their disability. The pain acceptance programs, such as the Acceptance Commitment Therapy [111], may tackle the chronic pain and further elevate the functional status as well as emotional and physical well-being [112,113]. Resilience intervention has also shown positive results in people with diabetes [114,115], after suffering a stroke [116] and as therapy for parents or other people who care for people with disabilities [117,118] or chronic illnesses [119]. In general, the results show the positive effects of resilience intervention on reducing stress and improving the ability to cope with stressful conditions, thus overall improving wellbeing and quality of life. However, some authors have noted concerns about universal resilience interventions [120], such as the possibility of focusing on the individual and overlooking environmental aspects. Therefore, it is necessary to provide resilience interventions adapted to the characteristics of the disability and the variables studied in applied research [121,122].

### 4.2. Limitations

This study has several limitations. First, our sample was voluntary in nature, which may imply different motivations to participate in the research. Second, the sample did not include all degrees of disability since the higher level was underrepresented. We did not consider additional diagnoses in the studied sample, such as depression, PTSD/trauma, and substance use, at the time of participation. These factors could affect stigma perception and consequently psychological quality of life. We also did not examine life and biomedical variables related to adaptation to the course of the disease [123].

Further research should use samples with different types, levels and grades of disability. Differences may be identified regarding the severity of the disability. It should also be considered whether a disability is congenital or acquired because this difference can produce different environmental adaptations to the disability. Longitudinal studies should be conducted to explore the mediating role of resilience over time to identify whether personal strengths evolve over the course of disability.

## 5. Conclusions

First, this study shows the negative association between internalized stigma and quality of life in people with physical disability. Second, our research reveals the mediator role of resilience in the relationship between internalized stigma and the psychological quality of life of people with physical disability. The results show that some interactive processes, such as resilience, may contribute to decreasing the negative effects of internalized stigma. Intervention programmes aimed to improve psychological quality of life of people with disability should promote resilience adapted to the characteristic of the disability.

## Figures and Tables

**Table 1:** Descriptive statistics and Pearson correlation coefficients for the variables in the study.

	*M*	*SD*	1	2	3	4	5
1. Internalized stigma	1.98	0.71	-	0.18 **	–0.01	–0.55 **	–0.51 **
2. Group identification	2.69	0.78		-	0.53 **	0.07	0.03
3. Collective action	3.16	0.69			-	0.23 **	0.13
4. Resilience	3.00	0.63				-	0.62 **
5. Psychological quality of life	3.34	0.55					-

*Notes.* Scores ranged from 1 to 4, except for psychological quality of life (which ranged from 1 to 5). ** p < 0.01.

**Table 2:** Results of mediational analysis.

Mediational Analysis		Direct Beta without Mediator	Direct Beta with Mediator	Indirect Beta [CI]
IE → GI and CA → Resilience	No mediation	−0.53 ***	−0.55 ***	0.02 ** [0.008 – 0.043]
IE → GI → CA	No mediation	−0.01	0.09 **	0.03 [0.036 – 0.158]
IE → Resilience → PQL	Partial mediation	−0.61 ***	−0.32 ***	−0.29* [−0.223 – −0.371]

Note: IE, Internalized Stigma; GI, Group Identification; CA, Collective Action; PQL, Psychological Quality of Life.
